# Clinical Remission of Severe Crohn’s Disease with Empagliflozin Monotherapy in a Pediatric Patient with Glycogen Storage Disease Type 1b

**DOI:** 10.1097/PG9.0000000000000356

**Published:** 2023-08-28

**Authors:** Lauren V. Collen, Peter E. Newburger, Scott B. Snapper

**Affiliations:** From the * Division of Gastroenterology, Hepatology, and Nutrition, Boston Children’s Hospital, Harvard Medical School, Boston, MA; † Departments of Pediatrics and Molecular, Cell, and Cancer Biology, UMass Chan Medical School, Worcester, MA; ‡ Dana-Farber/Boston Children’s Hospital Cancer and Blood Disorders Center, Harvard Medical School, Boston, MA.

**Keywords:** glycogen storage disease, inflammatory bowel disease, very early onset inflammatory bowel disease, Crohn’s disease, empagliflozin, SGLT2 inhibitor, precision medicine

## Abstract

Glycogen storage disease type 1b (GSD1b) is associated with inflammatory bowel disease and congenital neutropenia. Neutropenia in GSD1b is caused by the accumulation of 1,5-anhydroglucitol-6-phosphate. Empagliflozin is an antidiabetic drug that promotes renal excretion of this metabolite. We report on a patient with refractory GSD1b-associated inflammatory bowel disease who is in clinical remission on empagliflozin monotherapy.

## INTRODUCTION

Glycogen storage disease type 1b (GSD1b) is a rare disorder of glycogen metabolism and is associated with monogenic inflammatory bowel disease (IBD) and congenital neutropenia (1,2). GSD1b is caused by mutations in *SLC37A4*, which encodes glucose-6-phosphate translocase, an enzyme necessary for glycogenolysis and gluconeogenesis. GSD1b typically presents in infancy with hypoglycemia, hepatomegaly, and growth failure (2). Neutropenia and neutrophil dysfunction are also prominent features of GSD1b and are the drivers of IBD pathogenesis in this population (3). The mechanism of neutropenia and neutrophil dysfunction in GSD1b was recently discovered to be from the accumulation of the toxic metabolite 1,5-anhydroglucitol-6-phosphate (1,5AG6P) in neutrophil cytoplasm. 1,5AG6P inhibits hexokinases necessary for glycolysis, respiratory burst, and protein glycosylation, resulting in accelerated apoptosis and neutrophil dysfunction (4). Granulocyte colony-stimulating factor (G-CSF) has been a mainstay in the treatment of neutropenia-mediated features of GSD1b, including IBD, but, importantly, it does not address neutrophil dysfunction (3,5,6). Empagliflozin is a sodium-glucose co-transporter 2 (SGLT2) inhibitor approved for use in type 2 diabetes. SGLT2 inhibitors work by preventing renal re-absorption of 1,5-anhydroglucitol (1,5AG) (a precursor to 1,5AG6P), which in turn results in 4-fold decreases of 1,5AG in blood and of 1,5AG6P in neutrophils, improving neutrophil function and partially or completely correcting neutropenia (6). We describe the successful off-label use of empagliflozin in the treatment of severe, infliximab-refractory Crohn’s disease in a patient with GSD1b.

## CASE REPORT

**FIGURE 1.**
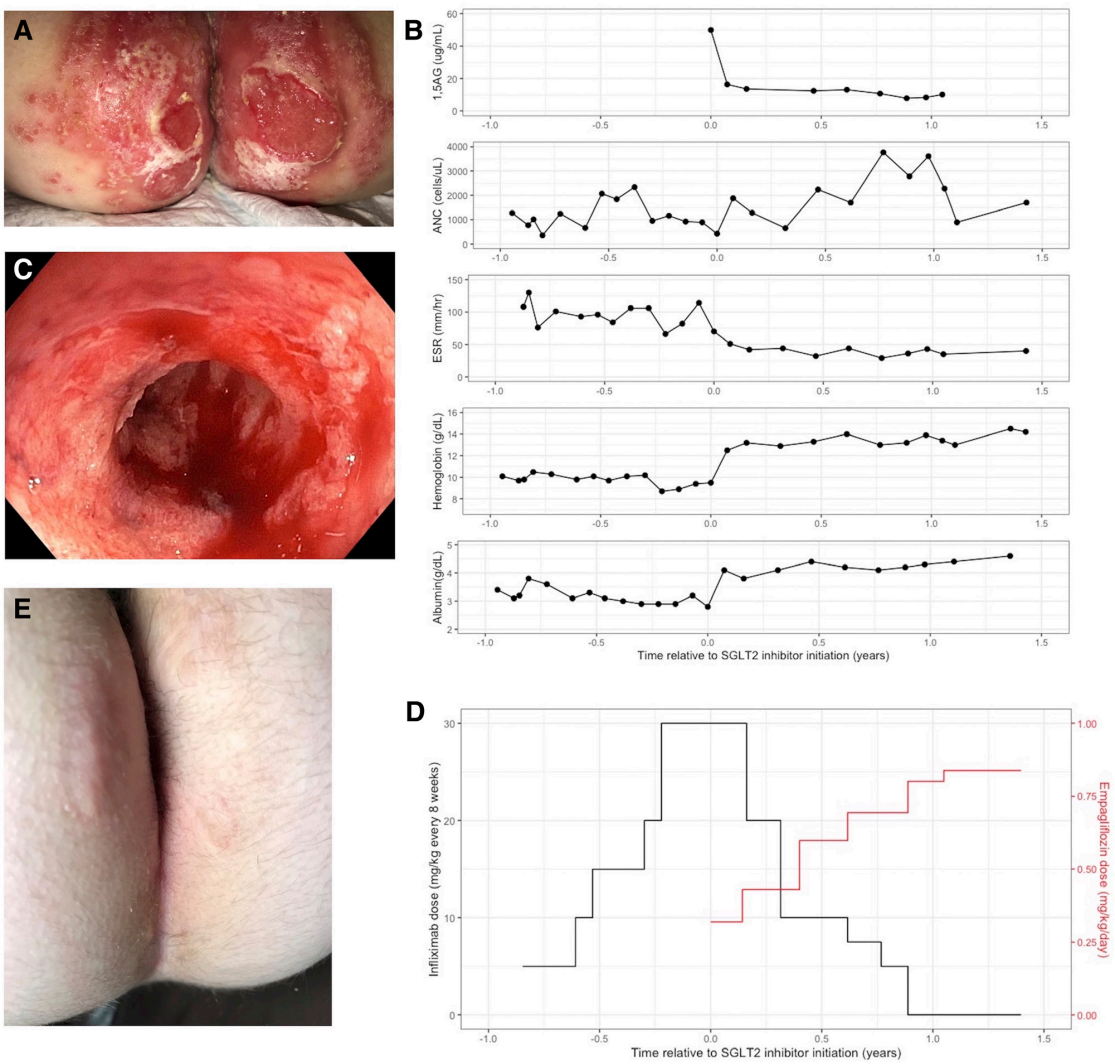
A) Deep ulcerations affecting buttocks and perianal region, with Candidal superinfection. B) Laboratory trend relative to empagliflozin initiation. Time 0 reflects the empagliflozin start date. There was a decrease in 1,5-anhydroglucitol (1,5AG) and erythrocyte sedimentation rate (ESR) and an increase in absolute neutrophil count (ANC), hemoglobin, and albumin after initiation of empagliflozin. C) Ascending colonic stricture. Inflammation is characterized by friability, edema, featureless appearance, and deep ulcerations. D) Infliximab escalation and de-escalation (black, left axis) relative to empagliflozin initiation and escalation (red, right axis). Infliximab dosing intervals varied from every 4 to 8 weeks, so is presented as the total dose per 8-week period. Following the initiation of empagliflozin, the infliximab dose was decreased and ultimately discontinued. E) Resolution of chronic ulcerations affecting buttocks and perianal region after initiation of empagliflozin.

A 10-year-old female with known GSD1b (*SLC37A4* c.818delG) and Crohn’s colitis treated since age 2 with mesalamine and G-CSF presented to care with symptoms of poorly controlled disease. Her symptoms included >15 episodes of bloody diarrhea per day, including frequent nocturnal stooling, severe abdominal pain, deep perianal ulcerations (Fig. 1A), severe fatigue, and a report of having been bedridden for >6 weeks. Laboratory evaluation was notable for markedly elevated inflammatory markers, anemia, and hypoalbuminemia (Fig. 1B). Pediatric Crohn’s Disease Activity Index (PCDAI) was 87.5, indicating severe disease activity. A colonoscopy revealed severe pancolitis with stricturing in the ascending colon (Fig. 1C). Histology demonstrated patchy, severely active colitis. She was induced with infliximab, and despite dose escalation to a maximum of 15 mg/kg every 4 weeks, she never achieved therapeutic levels. She reported transient symptomatic improvement after every infliximab infusion but never had sustained relief for the duration between doses and continued to have significantly elevated inflammatory markers, anemia, and hypoalbuminemia. After 1 year of persistent severe disease on high-dose infliximab, she was started on empagliflozin 5 mg twice daily (0.3 mg/kg/day), with rapid symptomatic response and improvement in laboratory data (Fig. 1B, D). Serum 1,5AG levels, which were above the upper limit of detection before the initiation of empagliflozin, normalized within 1 month. Within 3 months of empagliflozin initiation, she was in clinical remission, with the resolution of bloody diarrhea, abdominal pain, and perianal ulcerations (Fig. 1E) and markedly increased energy levels, reflected in the improvement in PCDAI to 12.5. By 18 months after initiation of empagliflozin, the dose had been gradually increased to 0.75 mg/kg/day and infliximab had been discontinued for over 6 months, with continuous clinical and biomarker remission (Fig. 1D), including normalization of stool calprotectin and lactoferrin. The patient was additionally able to discontinue G-CSF with stable neutrophil counts and no significant infections. Notably, while clinical remission of IBD was achieved and sustained shortly after empagliflozin initiation, higher dosing was needed to stabilize neutrophil counts and support G-CSF weaning. Dose escalation was carried out gradually given the known risk of hypoglycemia and the patient history of 2 hypoglycemic seizures which predated empagliflozin initiation. No adverse effects from empagliflozin were observed. Specifically, the patient had a continuous glucose monitor, and there were no clinically significant hypoglycemic events in the observation period. Endoscopic re-assessment for mucosal healing was recommended but declined by the patient’s family, as the previous endoscopy was complicated by severe aspiration pneumonia.

## DISCUSSION

We report on the successful off-label use of empagliflozin, an oral SGLT2 inhibitor, as a mechanism-based treatment of IBD in a pediatric patient with GSD1b. To our knowledge, this is the first report of a GSD1b patient with severe, infliximab-refractory IBD who was able to achieve clinical and biomarker remission and discontinue her biologic and G-CSF with empagliflozin as primary IBD-directed therapy. There are other reports in the literature describing the use of empagliflozin in the treatment of GSD1b, however, the focus of these reports has been on neutropenia and utilization of empagliflozin to support weaning of G-CSF in largely biologic-naïve patients (6–12). The first report of SGLT2 inhibitors in GSD1b by Wortmann et al. (6) described 4 patients with GSD1b and incomplete response to G-CSF; SGLT2 inhibitor initiation allowed for G-CSF discontinuation in 2 patients and dose lowering in 2 others. Three of these 4 patients were treated for IBD, but most, unlike our patient, had milder disease managed with G-CSF and sulfasalazine (6). Kaczor et al (7) described 4 patients with GSD1b, 3 of whom had IBD. All 3 had been managed with G-CSF and mesalamine and had improvement in stool number with empagliflozin initiation but no further IBD-specific assessment (7). There are additional case reports of empagliflozin use in individual patients with GSD1b and IBD, including in a 32-year-old who was able to decrease G-CSF and discontinue mesalamine (12); a 17-year-old who continued sulfasalazine but discontinued G-CSF (11); an 11-year-old who was able to decrease the dose of G-CSF (10); a 35-year-old with a history of subtotal colectomy and extensive small bowel resection with mild to moderate colitis and poor wound healing postoperatively who after initiation of empagliflozin had improved wound healing, discontinuation of G-CSF, but persistent mild IBD activity (8); and a 14-year-old with IBD and arthritis who initiated empagliflozin after adalimumab after antibody development with improvement in PCDAI but persistent elevations in calprotectin (9). The case presented in this report is unique in that our patient had severe intestinal disease refractory to the combination of G-CSF and infliximab, yet was able to sustain clinical and biomarker remission with empagliflozin monotherapy.

Beyond our patient’s dramatic response to empagliflozin and the appeal of a precision medicine approach, SGLT2 inhibitor use has additional advantages over conventional therapies. Most importantly, it has a favorable safety profile, with primary adverse effects being hypoglycemia and urinary tract infections (13), neither of which occurred in our patient. This is in contrast to G-CSF, which is associated with hypersplenism, bone pain, myelodysplastic syndrome, and acute myeloid leukemia (14), and immunosuppressants such as infliximab, which are associated with increased risks for opportunistic infections and lymphoma. Additionally, SGLT2 inhibitors are oral and significantly less costly than G-CSF and biologics. Overall, the remarkable efficacy of SGLT2 inhibition in this particularly refractory case of IBD in a child with GSD1b, in combination with its favorable side effect profile and ease of administration, make it an attractive treatment option for GSD1b patients.

## ACKNOWLEDGMENTS

The authors would like to thank our patient and her family, who provided informed consent for the publication of her experience.
